# Using Restricted Cubic Splines to Study the Trajectory of Systolic Blood Pressure in the Prognosis of Acute Myocardial Infarction

**DOI:** 10.3389/fcvm.2021.740580

**Published:** 2021-09-10

**Authors:** Shuai Zheng, Fengzhi Zhao, Rui Yang, Wentao Wu, Hui Liu, Wen Ma, Fengshuo Xu, Didi Han, Jun Lyu

**Affiliations:** ^1^Department of Clinical Research, The First Affiliated Hospital of Jinan University, Guangzhou, China; ^2^School of Public Health, Shaanxi University of Chinese Medicine, Xianyang, China; ^3^Intensive Care Unit, The First Affiliated Hospital of Jinan University, Guangzhou, China; ^4^School of Public Health, Xi'an Jiaotong University Health Science Center, Xi'an, China; ^5^School of Public Health, Lanzhou University, Lanzhou, China

**Keywords:** SBP, AMI, prognosis, non-linear, MIMIC

## Abstract

**Background:** Acute myocardial infarction (AMI) is still the most serious manifestation of coronary artery disease. Systolic blood pressure (SBP) is the best predictor of blood pressure in AMI. Thus, its influence on AMI is necessary to be explored.

**Methods:** A total of 4,277 patients with AMI were extracted from the Medical Information Mart for Intensive Care database. Chi-square test or Student's *t*-test was used to judge differences between groups, and Cox regression was used to identify factors that affect AMI prognosis. SBP was classified as low (<90 mmHg), normal (90–140 mmHg), or high (>140 mmHg), and a non-linear test was performed. Meaningful variables were incorporated into models for sensitivity analysis. Patient age was classified as low and high for subgroup analysis, and the cutoff value of the trajectory was identified. *P* < 0.05 indicates statistical significance.

**Results:** The effect of SBP on the prognosis of patients with AMI is non-linear. The risks in models 1–3 with low SBP are 6.717, 4.910, and 3.080 times those of the models with normal SBP, respectively. The risks in models 1–3 with high SBP are 1.483, 1.637, and 2.937 times those of the models with normal SBP, respectively. The cutoff point (95% confidence interval) of the trajectory is 114.489 mmHg (111.275–117.702 mmHg, all *P* < 0.001).

**Conclusions:** SBP has a non-linear effect on AMI prognosis. Low and high SBP show risks, and the risk of low SBP is obviously greater than that of high SBP.

## Introduction

The mortality and morbidity of acute myocardial infarction (AMI) are declining in most countries, especially in countries with higher per capita income (1–3). However, the prevalence of long-term AMI is also increasing with the aging of the world population and rapid population growth; thus, the disease burden of AMI is increasing (1). Every year, 2.4 million people die in the United States, and four million people die in Europe and North Asia; coronary artery disease (CAD) causes up to one-third of these deaths (4–7). AMI is the most serious manifestation of CAD and greatly increases the mortality rate of CAD (8).

The pathogenesis of acute coronary syndrome is a complex pathophysiological process accompanied by complex neuroendocrine changes (9). Neuroendocrine response after AMI results in the activation of the sympathetic nervous system and renin–angiotensin system and the release of vasopressin and atrial natriuretic peptide. The net effect of this response is vasoconstriction, cardiac stimulation, and regional flow redistribution, which may have a favorable effect in some situations and a deleterious effect in others. Therefore, blood pressure (BP) measurement can be used to reflect the overall potential performance of the cardiovascular and neuroendocrine systems after AMI (10).

As early as the 1990s, studies have shown that systolic blood pressure (SBP), diastolic blood pressure (DBP), and pulse pressure are risk factors for cardiovascular disease (11). A study in 2000 showed that the average values of SBP, DBP, and mean blood pressure (MBP) are important predictors of cardiovascular disease in young men (<60 years old), and mean SBP and pulse pressure are important predictors in elderly men (>60 years old) (12). In 2015, Sundström and Arima found that SBP and DBP are very important independent risk factors for cardiovascular and renal diseases (13). The various indicators of BP have played an irreplaceable warning role in the treatment and prognosis of cardiovascular diseases.

Admission SBP can be used as a predictor of rapid clinical evaluation and poor cardiovascular prognosis studies. If this indicator can be effectively used to assess the risk of adverse consequences, then a treatment plan for patients with new AMI can be quickly developed (14). Therefore, a more comprehensive and detailed understanding of the performance of SBP in the prognosis of AMI is important. The purpose of this study was to use restricted cubic splines to study the performance of SBP in patients with AMI from the Medical Information Mart for Intensive Care (MIMIC) database.

## Materials and Methods

### Patients and Variables

**Figure 1:**
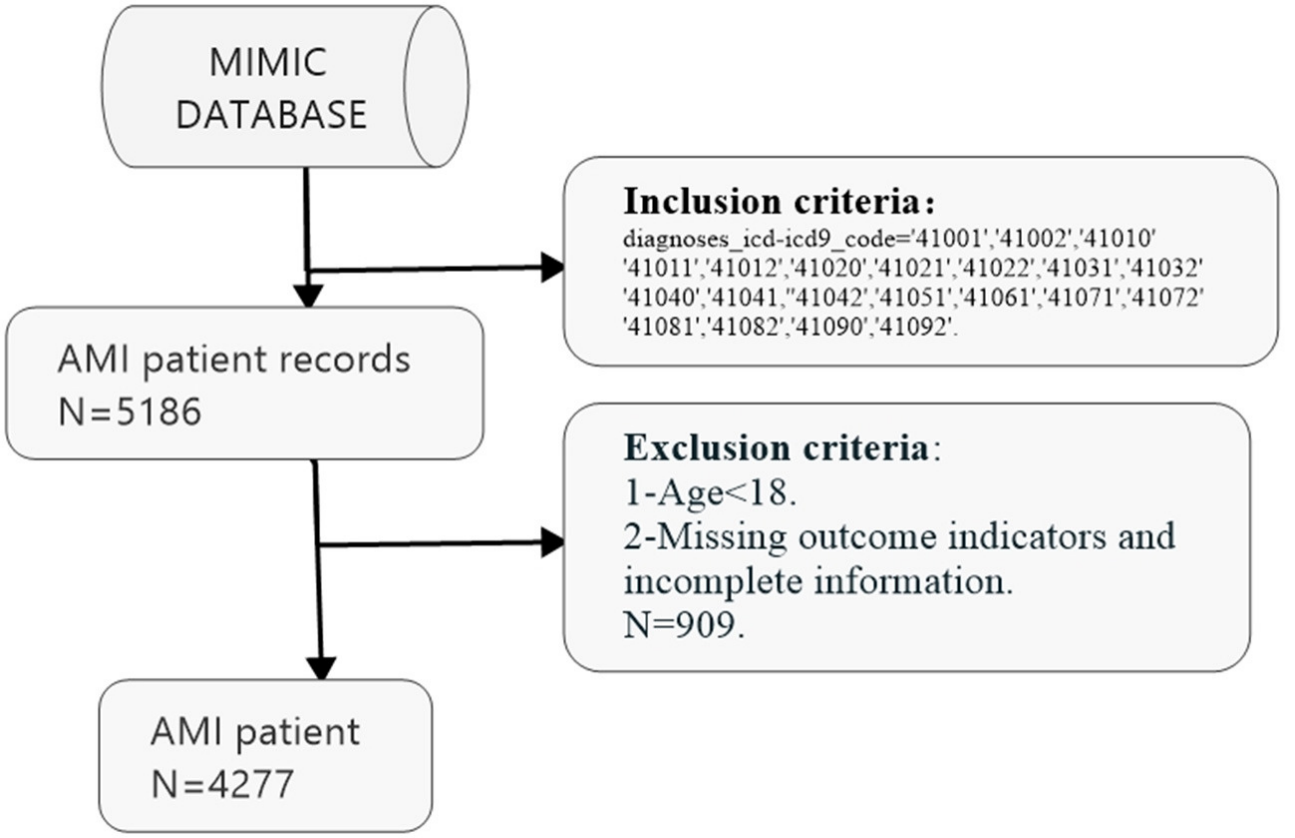
The inclusion and exclusion criteria of this study.

The MIMIC [database jointly issued by the Massachusetts Institute of Technology (MIT) Computational Physiology Laboratory, Beth Israel Dikang Medical Center, and Philips Medical] is supported by the National Institutes of Health to promote the work of intensive medical research (15). MIMIC is a publicly available dataset developed by the MIT Computational Physiology Laboratory that includes unidentified patient health data related to ~60,000 intensive care unit visits. The dataset includes demographic information, vital signs, laboratory tests, drugs, and other information (16). The database has a large number of samples, comprehensive information, and long-term patient tracking; can be used for free; and provides a wealth of resources for intensive care research (17). Access to the database (Certificate Number: 38489997) was granted after the completion of the National Institutes of Health's web-based training course, “Protecting Human Research Participants.” In the present study, we extracted 4,277 AMI data from the MIMIC database. All patients were diagnosed with AMI for the first time upon admission. The following variables were extracted from the information of the patients with AMI in the MIMIC database: gender, atrial fibrillation, atrial flutter (AFL), ventricular fibrillation (VF), ventricular tachycardia (VT), SBP, drug, total calcium level, chloride level, creatinine level, phosphate level, potassium level, sodium level, nitrogen level, hemoglobin count, platelet count, red blood cell width (RDW), white blood cell (WBC) count, age, respiration rate, mean heart rate, mean BP, DBP, mean glucose level, Sequential Organ Failure Assessment (SOFA) score, and Acute Physiology Score (APS)-III when the patient was admitted to the hospital. Survival time (in months) and status were also extracted from the database. The inclusion and exclusion criteria are shown in Figure 1.

### Statistical Analysis

The patients were divided into two groups according to life and death outcomes. In addition to status and survival time, the chi-square test was performed on categorical variables, and Student's *t*-test was performed on continuous variables. Then, Cox regression was performed on all variables to explore the variables that have an impact on AMI outcome. Finally, SBP was categorized as low (<90 mmHg), normal (90–140 mmHg), and high (>140 mmHg). A non-linear test was performed to judge whether the effect of SBP on AMI prognosis is non-linear. The patients were also divided into the “advanced age” and “low age” groups for subgroup analysis. Meaningful variables were incorporated in the Cox regression results to construct three models, and then sensitivity analysis was performed on the models. SBP alone was incorporated in model 1; some variables were collectively included in model 2, and all meaningful variables were included in the Cox regression result as model 3. The best cutoff point for the entire prognostic trajectory was determined. *P* < 0.05 was considered statistically significant. Statistical analyses were conducted using Excel, SPSS, and R (ggplot2, rms, survival, and segmented packages).

## Results

Among the 4,277 patients with AMI, 843 died during follow-up. According to our results, gender (*P* = 0.324) may not be a factor affecting AMI outcome. The proportion of male/female (%) in the survival group was 2,200/1,234 (64.1%/35.9%), and that in the death group was 556/287 (66.0%/34.0%). The mean value [95% confidence interval (95% CI)] of the platelet count (*P* = 0.347) of the same patients in the survival group was 236.00 (188.00–294.00), whereas the mean value (95% CI) in the death group was 233.00 (173.00–308.50). The incidence of AFL in the survival and death groups was 2.3% (*N* = 79) and 2.4% (*N* = 20, *P* = 1), respectively. All variables except gender (*P* = 0.324), AFL (*P* = 1), and platelet (*P* = 0.347) showed differences in AMI outcome. The results of the chi-square test and Student's *t*-test are shown in Table 1.

**Table 1:** Baseline characteristics of patients in the study.

**Variable**	**Live**	**Dead**	***P*-value**
*N*	3,434	843	
Sex			0.324
Male	1,234 (35.9)	287 (34.0)	
Female	2,200 (64.1)	556 (66.0)	
AF			<0.001
Yes	931 (27.1)	323 (38.3)	
No	2,503 (72.9)	520 (61.7)	
AFL			1
Yes	79 (2.3)	20 (2.4)	
No	3,355 (97.7)	813 (97.6)	
VF			0.01
Yes	139 (4.0)	52 (6.2)	
No	3,295 (96.0)	791 (93.8)	
VT			<0.001
Yes	281 (8.2)	105 (12.5)	
No	3,153 (91.8)	738 (87.5)	
Drug			0.001
Yes	1,829 (53.3)	395 (46.9)	
No	1,605 (47.7)	448 (53.1)	
Total_Ca	8.7 (8.2–9.1)	8.5 (7.9–9.0)	<0.001
Chloride	103.0 (100.0–106.0)	102.0 (98.0–106.0)	<0.001
Creatinine	1.0 (0.8–1.5)	1.5 (1.0–2.4)	<0.001
Phosphate	3.5 (2.9–4.1)	3.9 (3.2–5.0)	<0.001
Potassium	4.2 (3.8–4.6)	4.4 (3.9–4.9)	<0.001
Sodium	139.0 (136.0–141.0)	138.0 (135.0–141.0)	0.009
Nitrogen	21.0 (15.0–31.0)	33.0 (21.0–51.0)	<0.001
Hemoglobin	12.2 (10.6–13.7)	11.2 (9.9–12.5)	<0.001
Platelet	236.0 (188.0–294.0)	233.0 (173.0–308.5)	0.347
RDW	13.8 (13.1–14.9)	14.9 (13.7–16.7)	<0.001
WBC	10.4 (8.0–13.7)	12.3 (8.9–16.7)	<0.001
Age	69.0 (58.3–78.0)	77.0 (68.0–83.0)	<0.001
Respiration rate	25.0 (18.5–29.0)	28.0 (24.0–33.0)	<0.001
Mean HR	80.7 (71.2–90.1)	86.2 (75.1–97.7)	<0.001
MBP	58.0 (51.8–83.0)	53.0 (46.0–61.0)	<0.001
DBP	58.8 (53.0–65.7)	55.2 (49.5–62.0)	<0.001
SBP	113.5 (105.4–124.2)	108.5 (99.5–122.0)	<0.001
Mean glucose	134.8 (117.2–163.0)	149.0 (121.3–187.1)	<0.001
SOFA	3.0 (1.0–5.0)	6.0 (4.0–9.0)	<0.001
APS-III	37.0 (28.0–49.0)	55.0 (45.0–72.0)	<0.001

*AF, atrial fibrillation; AFL, atrial flutter; VF, ventricular fibrillation; VT, ventricular tachycardia; RDW, red blood cell width; WBC, white blood cell count; Mean HR, mean heart rate; SBP, systolic blood pressure; MBP, mean blood pressure; DBP, diastolic blood pressure; SOFA, Sequential Organ Failure Assessment; APS-III, Acute Physiological Score*.

All variables were incorporated into the Cox regression of model 3. The results showed that age, VF, VT, drug, total calcium level, chloride level, creatinine level, phosphate level, platelet count, RDW, WBC count, respiration rate, mean heart rate, mean glucose, SOFA score, and APS-III score are the prognostic factors of AMI (all *P* < 0.001, Table 2).

**Table 2:** The results of cox regression analysis.

**Variable**	**HR**	**95% CI**	***P*-value**
Age	1.0477	1.0382–1.0572	<0.001
VFI			<0.001
No	Reference		
Have	1.9324	1.3678–2.7302	
VT			<0.001
No	Reference		
Have	1.589	1.2232–2.0642	
Drug			0.027
No	Reference		
Have	0.8275	0.6999–0.9784	
Total_Ca	0.8470	0.7668–0.9357	0.001
Chloride	0.9721	0.9523–0.9922	0.007
Creatinine	0.9318	0.8817–0.9847	0.012
Phosphate	1.1209	1.0522–1.1940	<0.001
Platelet	0.9991	0.9984–0.9998	0.011
RDW	1.1483	1.1014–1.1972	<0.001
WBC	1.0115	1.0043–1.0188	0.002
Respiration rate	1.0095	1.0006–1.0185	0.036
Mean HR	1.0106	1.0044–1.0169	<0.001
Mean glucose	1.0035	1.0021–1.0049	<0.001
SOFA	1.0488	1.0106–1.0883	0.012
APS-III	1.0219	1.0162–1.0276	<0.001

**Figure 2:**
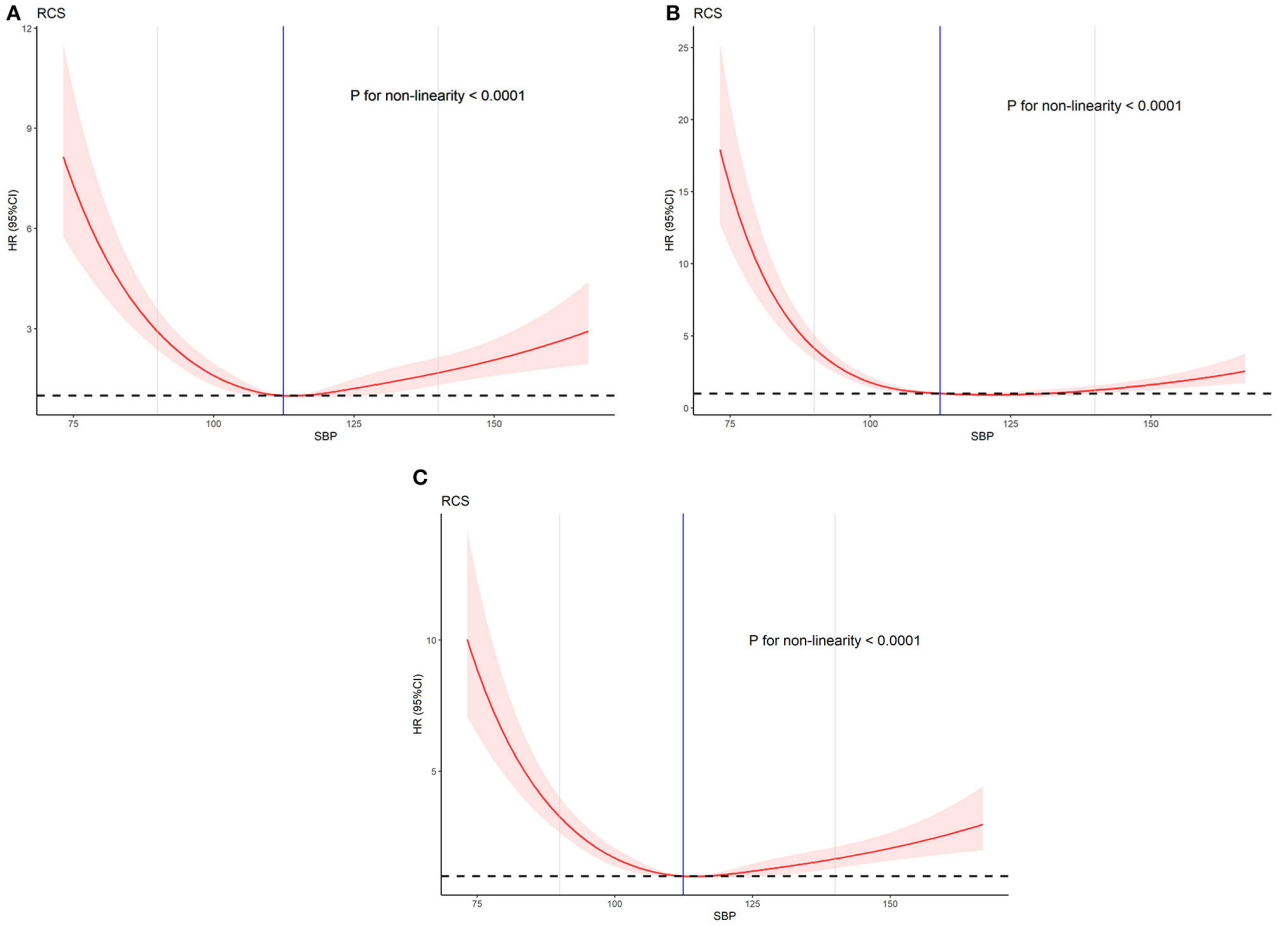
The effect of different doses of SBP on the prognosis of AMI. **(A)** Univariate. **(B)** Adjusted for age, VF, VT, drug, SOFA, and APS-III. **(C)** Adjusted for age, VF, VT, Total_Ca, chloride, creatinine, phosphate, platelet, RDW, WBC, respiration rate, mean HR, mean glucose, drug, SOFA, and APS-III.

The statistical results of the RCS test are shown in Table 3. Model 1 incorporates a single variable (SBP) into the analysis as shown in Figure 2A. The hazard ratio (HR) with (95% CI) of model 1 with low SBP is 6.717 (5.044–8.946, *P* < 0.001), and that of model 1 with high SBP is 1.483 (1.120–1.965, *P* < 0.01). Model 2 incorporates age into the analysis as shown in Figure 2B. The HR of model 2 with low SBP is 4.910 (3.679–6.552, *P* < 0.001), and that of model 2 with high SBP is 1.637 (1.231–2.18, *P* < 0.001). Model 3 incorporates all meaningful factors in all Cox regression results into the analysis as shown in Figure 2C. The HR (95% CI) of model 3 with low SBP is 3.080 (2.162–4.385, *P* < 0.001), and that of model 2 with high SBP is 2.937 (1.918–4.498). SBP is non-linear in the prognosis curves of AMI in all models. The trend of the curve changed at about SBP = 110 mmHg (Figure 2, all *P* < 0.001). Notably, the HRs of all models with low SBP are greater than those with high SBP (*P* < 0.05). This finding indicates that the risk of patients with AMI who have SBP <90 mmHg is higher than that of patients with SBP >140 mmHg. This performance becomes more obvious and statistically significant with the addition of more adjustment variables.

**Table 3:** Cox regression analyses of the relationship between SBP and AMI prognosis.

**Variable**	**Model 1**		**Model 2**		**Model 3**	
	**HR (95% CI)**	***P*-value**	**HR (95% CI)**	***P*-value**	**HR (95% CI)**	***P*-value**
Normal	1		1		1	
Low	6.717 (5.044–8.946)	<0.001	4.910 (3.679–6.552)	<0.001	3.080 (2.162–4.385)	<0.001
High	1.483 (1.120–1.965)	0.01	1.637 (1.231–2.18)	<0.001	2.937 (1.918–4.498)	<0.001

*Model 1: univariate. Model 2: adjust for aged, VF, VT, drug, SOFA, and APS-III. Model 3: adjusted for age, VF, VT, Total_Ca, chloride, creatinine, phosphate, platelet, RDW, WBC, respiration rate, mean HR, mean glucose, drug, SOFA, and APS-III*.

**Figure 3:**
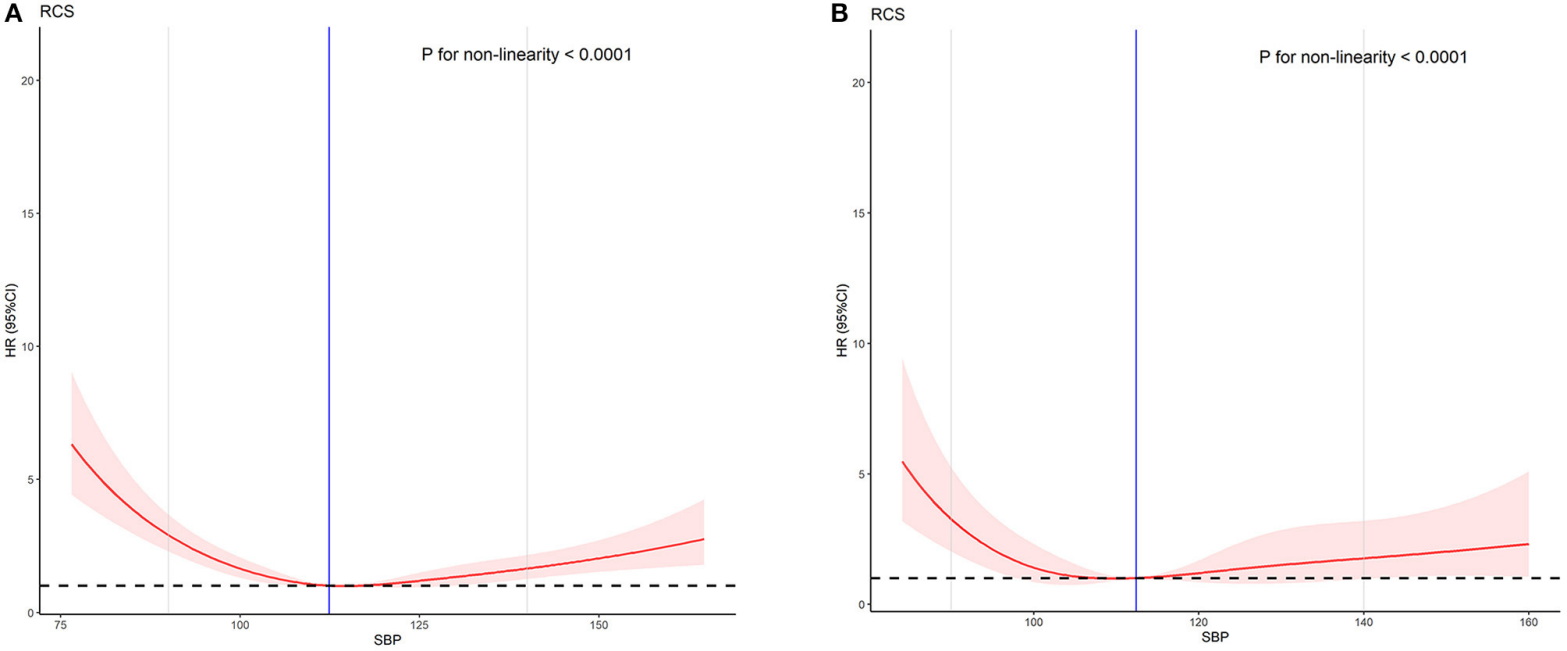
The effect of SBP on the prognosis of AMI between different age groups. **(A)** Low-age group (age ≤ 65). **(B)** High-age group (age > 65).

Age is a prognostic factor of AMI in the Cox regression analysis results. Therefore, we divided the patients into the low-age (≤65 years) and high-age groups (>65 years) and performed an age subgroup analysis. The baseline characteristics and COX regression analysis results of the subgroups are shown in Table 4 and Table 5. The results of the two groups are all non-linear (*P* < 0.001). The non-linear curve performance of the two groups is shown in Figure 3. The slope of the low SBP part is steeper than the slope of the high SBP part. This result reflects that the risk of low SBP is higher than that of high SBP in patients with AMI. The results are similar to the performance of all models.

**Table 4:** Baseline characteristics between different age groups.

**Variable**	**Low-age group**	**High-age group**	***P-*value**
	**(age < 65)**	**(age > 65)**	
*n*	1,616	2,661	
Status			<0.001
Dead	159 (9.8)	684 (25.7)	
Live	1,457 (90.2)	1,977 (74.3)	
Sex			0.01
Male	614 (38.0)	907 (34.1)	
Female	1,002 (62.0)	1,754 (65.9)	
AFI			<0.001
Yes	253 (15.7)	1,001 (37.6)	
No	1,363 (84.3)	1,660 (62.4)	
AFL			0.01
Yes	24 (1.5)	75 (2.8)	
No	1,592 (98.5)	2,586 (97.2)	
VFI			0.001
Yes	95 (5.9)	96 (3.6)	
No	1,521 (94.1)	2,565 (96.4)	
VT			0.01
Yes	1,445 (89.4)	215 (8.1)	
No	171 (10.6)	2,446 (91.9)	
SBP			0.64
Normal	1,472 (91.1)	2,403 (90.3)	
Lower	38 (2.4)	73 (2.7)	
Higher	106 (6.6)	185 (7.0)	
Drug			0.28
Yes	858 (53.1)	1,366 (51.3)	
No	758 (46.9)	1,295 (48.7)	
Total_Ca	8.7 (8.1–9.1)	8.6 (8.1–9.1)	0.83
Chloride	103.0 (100.0–106.0)	103.0 (100.0–106.0)	0.11
Creatinine	1.0 (0.8–1.4)	1.2 (0.9–1.7)	<0.001
Phosphate	3.5 (2.9–4.2)	3.6 (3.0–4.3)	<0.001
Potassium	4.2 (3.8–4.6)	4.2 (3.8–4.7)	<0.001
Sodium	138.0 (136.0–140.0)	138.0 (136.0–141.0)	0.06
Nitrogen	18.0 (14.0–27.0)	25.0 (18.0–39.0)	<0.001
Hemoglobin	12.8 (11.0–14.2)	11.6 (10.2–13.0)	<0.001
Platelet	245.0 (196.0–299.0)	230.0 (180.0–295.0)	<0.001
RDW	13.7 (13.0–14.7)	14.2 (13.4–15.5)	<0.001
WBC	11.0 (8.3–14.4)	10.5 (8.0–14.2)	0.66
Respiration rate	25.0 (17.5–29.0)	26.0 (23.0–30.0)	<0.001
Mean HR	82.5 (72.6–92.5)	81.2 (71.8–91.2)	0.02
Mean BP	61.0 (54.0–87.0)	55.0 (49.0–76.2)	<0.001
Mean glucose	134.1 (117.0–164.3)	139.1 (118.5–168.8)	0.01
SOFA	3.0 (1.0–5.3)	4.0 (2.0–7.0)	<0.001
APS-III	34.0 (25.0–48.0)	44.0 (34.0–57.0)	<0.001

**Table 5:** Cox regression results for different age groups.

**Variable**	**High-age group (age > 65)**			**Low-age group (age < 65)**		
	**HR**	**95% CI**	***P***	**HR**	**95% CI**	***P***
Age	1.0496	1.0335–1.0659	<0.001	1.0307	1.00045–1.0618	0.047
**VFI**						
No	Reference					
Have	1.8323	1.1856–2.8319	0.006	2.2974	1.21526–4.3433	0.01
**VT**						
No	Reference					
Have	1.4291	1.0698–1.9090	0.016	2.7406	1.63084–4.6056	<0.001
**Drug**						
No	Reference					
Have	0.8316	0.6931–0.9979	0.047	0.955	0.65098–1.4009	0.814
**SBP**						
Normal	Reference					
Lower	3.2289	2.2317–4.6718	<0.001	4.6389	2.46920–8.7150	<0.001
Higher	1.8212	1.2879–2.5754	<0.001	1.199	0.51073–2.8146	0.677
Total_Ca	0.8928	0.7994–0.9971	0.044	0.9793	0.81501–1.1766	0.823
Chloride	0.9814	0.9581–1.0053	0.127	0.9361	0.89230–0.9821	0.007
Creatinine	0.9093	0.8432–0.9806	0.014	0.8397	0.73948–0.9534	0.007
Phosphate	1.2223	1.1329–1.3188	<0.001	1.0415	0.90282–1.2016	0.577
Platelet	0.9987	0.9979–0.9995	0.001	1.0001	0.99855–1.0017	0.864
RDW	1.1476	1.0962–1.2014	<0.001	1.2934	1.17824–1.4197	<0.001
WBC	1.0258	1.0156–1.0362	<0.001	1.0017	0.98456–1.0192	0.845
Respiration rate	1.0171	1.0068–1.0275	0.001	1.022	1.00210–1.0422	0.03
Mean HR	1.007	1.0003–1.0139	0.041	1.0229	1.01017–1.0357	<0.001
Mean glucose	1.0025	1.0008–1.0042	0.003	1.0001	0.99600–1.0042	0.972
SOFA	1.0458	1.0037–1.0896	0.033	1.171	1.07203–1.2791	<0.001
APS-III	1.0198	1.0134–1.0262	<0.001	1.0185	1.00570–1.0314	0.004

**Figure 4:**
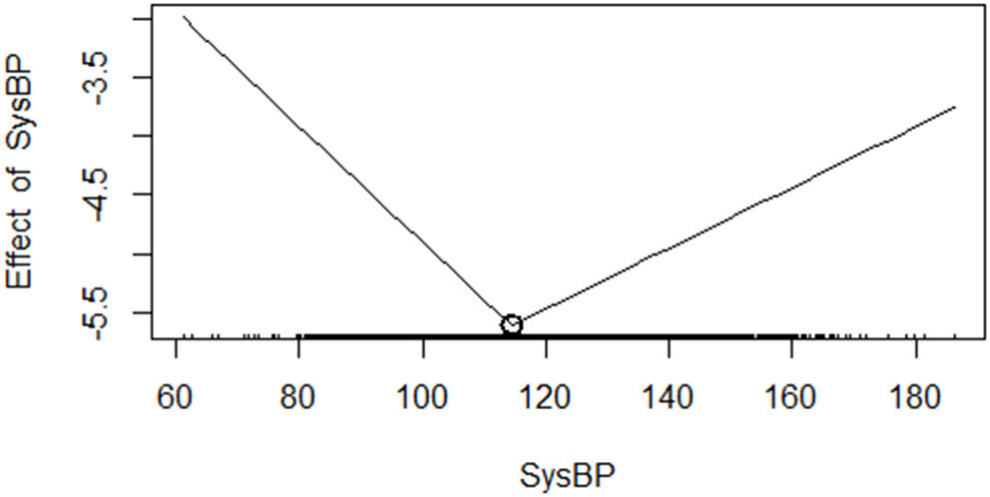
Identification of the trajectory cutoff value.

After the prognostic trajectory of SBP was verified as non-linear, a two-line piecewise linear model with a single change point was estimated by trying all possible values for the change point and choosing the value with the highest likelihood. The result showed that the cutoff point is 114.489 mmHg (111.275–117.702 mmHg). The HR (95% CI) of line 1 (SBP < 114.489 mmHg) is 0.952 (0.943–0.962, *P* < 0.001). This result means that for every 1 mmHg reduction in SBP, the risk of death in patients with AMI is reduced by 4.8% (1–0.952). The HR (95% CI) of line 2 (SBP > 114.489 mmHg) is 1.026 (1.018–1.035, *P* < 0.001), which also means that the risk of death in AMI patients increases by 2.6% (1.018–1) for every 1 mmHg increase in SBP (Figure 4).

## Discussion

In the past few decades, mortality from cardiovascular disease has been greatly reduced (18) partly because of the improved management of AMI (19). However, AMI is the most serious manifestation of CAD and the main cause of global cardiovascular disease morbidity and mortality (6, 20). A number of studies have pointed out that SBP is related to the prognostic risk of AMI (11–13, 21, 22). MBP may also be an important predictor. Pulse pressure has a little effect on the risk of cardiovascular disease; thus, SBP should be used in long-term monitoring as an indicator of AMI prognosis (23). Psaty and others pointed out that although DBP, SBP, and pulse pressure are directly related to the risk of coronary and cerebrovascular events, SBP is the best single predictor of cardiovascular events (22).

The results of our Cox multivariate analysis study showed that SBP (*P* < 0.001) is an influencing factor for AMI prognosis. All the prognostic curves of SBP in the RCS shows very similar performance. Our study sets a normal value range (90–140 mmHg) based on the normal physiological values in the human body, and the risk of AMI is the lowest within the normal value range. Notably, the performance of SBP in the prognosis of AMI in the RCS analysis is non-linear, and the non-linear test is statistically significant (*P* < 0.001). Moreover, in the sensitivity analysis, all models with low SBP had higher risks than those with high SBP. The risks in models 1–3 with low SBP are 6.717, 4.910, and 3.080 times those with normal SBP, and the risks in models 1–3 with high SBP are 1.483, 1.637, and 2.937 times those with normal SBP, respectively. In the subgroup analysis, the low-age and high-age groups also showed a higher risk among patients with low SBP compared with those with high SBP.

When the SBP of a patient with AMI is low to a certain level, the blood supply to the coronary artery becomes obstructed, which leads to myocardial ischemia, myocardial hypoxia, degeneration and necrosis, and eventually myocardial infarction. Several observational studies have shown that lowering the BP below a certain threshold may be harmful as reflected by the “J curve phenomenon” (24–26). An observational study of 22,672 patients with stable CAD showed that hypertension, low SBP (<120 mmHg), and low DBP (<70 mmHg) are all related to the heart. The increased risk of vascular events supported the “J curve phenomenon”; thus, low BP may be harmful in patients with coronary heart disease (27). Shiraishi et al. found that patients with AMI who have SBP <106 mmHg often reach Killip ≥3 upon hospital admission. The right coronary artery and left main trunk or multivessels are the culprits, the number of diseased blood vessels increases, and the hospital mortality rate increased considerably (28). The results of the Israeli Acute Coronary Syndrome Survey showed that SBP is related to cardiovascular events and total mortality. Patients with SBP <110 mmHg have a remarkably higher 1-year mortality risk ratio within 7 days (HR = 2.37) compared with those with normal SBP (110–140 mmHg, HR = 1.92) upon admission (29). Our study determined that the cutoff value of the SBP prognostic curve is 114.489 mmHg, and we quantified the risk of the entire curve. The result means that for every 1 mmHg reduction in SBP before 114.489 mmHg, the risk of death in patients with AMI is reduced by 4.8% (1–0.952). Moreover, the risk of death among patients with AMI increases by 2.6% (1.018–1) for every 1 mmHg increase in SBP after 114.489 mmHg. Lewington et al. also pointed out that SBP = 115 mmHg is an important observation threshold (30, 31). This value is very close to our findings.

SBP can reflect cardiac output and systemic peripheral resistance. A higher SBP at admission may indicate increased systemic resistance, maintenance of cardiac function, and less myocardial damage in patients with AMI. Metabolic syndrome refers to the co-occurrence of several known cardiovascular risk factors, including insulin resistance, obesity, atherogenic dyslipidemia, and hypertension. These conditions are interrelated and share underlying mediators, mechanisms, and pathways (32). Abnormal metabolism and blood pressure characterized by metabolic syndrome are risk factors for cardiovascular disease (33), particularly in young patients with AMI. These patients present extensive atherosclerotic disease in angiographic studies (34). Average arterial BP levels and short-term BP variability are related to hypertension-mediated organ damage, increased carotid intima-media thickness (35), and hypoperfusion, which further increase the risk of adverse events in AMI. The short-term outcomes of patients with normal SBP and with high admission SBP are similar, but with the passage of time, the outcome of patients with excessively elevated SBP upon admission is death, and the probability of major adverse cardiac events has an upward trend (9). Although different studies set different standards for normal BP, the results obtained are consistent, that is, patients with low SBP have a higher risk compared with patients with high SBP. Future studies would be interesting to explore the optimal blood pressure cutpoint by other methods, such as ambulatory blood pressure monitoring or home blood pressure measurement (36, 37).

## Conclusion

The risk of patients with AMI who have low SBP is very high; therefore, maintaining a high degree of vigilance for patients with AMI and low SBP is necessary. Continuous BP monitoring is particularly important; a normal SBP can guarantee a good prognosis for AMI, and high SBP has a certain low risk. The SBP value of 114.489 mmHg is the inflection point of the prognostic trajectory of AMI. Patients with AMI have the lowest probability of death when the SBP value is 114.489 mmHg. Continuous in-depth research on SBP is very necessary. SBP has a non-linear performance in AMI prognosis. Thus, the establishment of an accurate and reliable prognostic model that uses SBP to predict AMI has still a long way to go. We will further study the impact of SBP on AMI and strive to establish a prediction model with accurate threshold, high sensitivity, and reliability to guide clinicians and assist in decision-making.

## Limitations

First, our data were obtained from the MIMIC database. Most of the patients included in this database are from the United States; therefore, the generalization of the conclusion is limited. Second, parts of the index information of patients were not fully recorded, which led to the loss of information in the study. Finally, this study is a retrospective study, which may inevitably have some biases.

## Data Availability Statement

The original contributions presented in the study are included in the article/supplementary material, further inquiries can be directed to the corresponding author.

## Author Contributions

SZ: resources, investigation, validation, writing—original draft, and writing—review and editing. FZ: resources, investigation, validation, and writing—review and editing. RY: resources, investigation, and writing—review and editing. WW: resources, investigation, and formal analysis. HL, WM, FX, and DH: data curation and formal analysis. JL: project administration, supervision, and visualization. All authors contributed to the article and approved the submitted version.

## Conflict of Interest

The authors declare that the research was conducted in the absence of any commercial or financial relationships that could be construed as a potential conflict of interest.

## Publisher's Note

All claims expressed in this article are solely those of the authors and do not necessarily represent those of their affiliated organizations, or those of the publisher, the editors and the reviewers. Any product that may be evaluated in this article, or claim that may be made by its manufacturer, is not guaranteed or endorsed by the publisher.
